# Defective interfering virus protects elderly mice from influenza

**DOI:** 10.1186/1743-422X-8-212

**Published:** 2011-05-09

**Authors:** Paul D Scott, Bo Meng, Anthony C Marriott, Andrew J Easton, Nigel J Dimmock

**Affiliations:** 1 School of Life Sciences, University of Warwick, Coventry CV4 7AL, UK

**Keywords:** elderly, defective-interfering virus, geriatric, influenza, mice, treatment

## Abstract

**Background:**

We have identified and characterised a defective-interfering (DI) influenza A virus particles containing a highly deleted segment 1 RNA that has broad-spectrum antiviral activity. In young adult mice it exerts protection against several different subtypes of influenza A virus (defined here as homologous or genetically compatible protection) and against a paramyxovirus and an influenza B virus (heterologous or genetically unrelated protection). Homologous protection is mediated by replication competition between the deleted and full-length genomes, and heterologous protection occurs through stimulation of innate immunity, especially interferon type I.

**Methods:**

A single dose of the protective DI virus was administered intranasally to elderly mice at -7, -1 and +1 days relative to intranasal challenge with influenza A virus.

**Results:**

A single dose of the DI virus given 1 or 7 days protected elderly mice, reducing a severe, sometimes fatal disease to a subclinical or mild infection. In contrast, all members of control groups treated with inactivated DI virus before challenge became extremely ill and most died. Despite the subclinical/mild nature of their infection, protected mice developed solid immunity to a second infectious challenge.

**Conclusions:**

The defective interfering virus is effective in preventing severe influenza A in elderly mice and may offer a new approach to protection of the human population.

## Background

As a result of improved standards in the home, workplace, nutrition, and healthcare, the elderly are becoming an ever increasing proportion of the world's population. Yet because of waning immune competence and social factors, such as increased risk of infection in the communities in which many of them live, the elderly are vulnerable to a variety of infections, and particularly those that cause respiratory disease [1-4]. Elderly people exhibit a range of immune deficiencies that can be manifested in many facets of the immune response including T cell activation, CD4^+ ^and/or CD8^+ ^T cell activity, cytotoxic T cell activity, IL-2 secretion, antibody production and/or avidity, dendritic cells and type I interferon production [2,5-15]

Many of the causative agents of the most important respiratory diseases are viral, and amongst these influenza viruses and human respiratory syncytial virus (HRSV) are pre-eminent [16,17]. These agents are highly contagious and cause outbreaks in domestic communities with a high level of morbidity and often mortality. Since 1977 two subtypes of influenza A virus (H1N1 and H3N2) and an influenza B virus have been responsible for seasonal influenza. Pandemic 2009 H1N1 influenza entered the mix in April 2009 and appears to have replaced seasonal H1N1 virus. The surface haemagglutinin and neuraminidase antigens of these viruses evolve continuously so that immunity gained from infection becomes redundant after approximately 4 years, leaving individuals susceptible to further infection [18]. Thus the seasonal vaccine has to be formulated to match as far as possible the influenza A and B virus strains that are forecast to be circulating in 6 months' time. Efficacy of the vaccine depends, *inter alia*, on immune competence which declines in old age, and on providing the vaccination several weeks before the virus is encountered to allow the immune response time to mature [19-21]. However, a recent meta-analysis has questioned the efficacy of influenza vaccines in the over-65s [22]. The influenza antivirals Tamiflu and Relenza that are now available are most effective when given before or as soon as possible after infection, but solid evidence for their usefulness and safety in the elderly has not been found [23]. Resistance to Tamiflu, the more widely used antiviral, is already widespread in seasonal H1N1 virus [24-26].

In attempting to tackle respiratory disease we have pioneered a novel approach that exploits a naturally occurring influenza A antiviral that is made by the virus itself. This is a specific defective-interfering (DI) virus that confers protection from infection *in vivo*. Its active principle is a highly deleted version of the viral genomic RNA which acts to inhibit productive infection [27-31]. Influenza (*Orthomyxoviridae*) DI viruses were the first to be recognised [32], and have been studied extensively [30,33]. However analysis is difficult as natural DI preparations contain many different DI sequences. In part this is because its single-stranded, negative sense RNA genome exists as 8 discrete segments and DI RNAs can probably arise from any segment. While they are formed mainly from the three largest virion RNAs, any one virion RNA can give rise to many different DI RNA sequences through variably located central deletions [34,35]. We solved this heterogeneity problem by producing cloned viruses that contain one major species of DI RNA, and have characterized one DI virus, containing the 244 DI RNA derived from virion segment 1 of A/Puerto Rico/8/34 (PR8, H1N1), that is particularly active in protecting mice from a variety of different influenza A virus subtypes [36]. We call DI viruses that have demonstrable *in vivo *activity 'protecting viruses' [37]. Our protecting 244 RNA is encapsidated into authentic influenza virus particles, and these target the protecting RNA to cells that can be potentially infected by influenza virus.

Thus far we have established the efficacy of protecting virus by infecting young adult mice (approximately 5 weeks-old) representing several different inbred strains. Here we have investigated the antiviral efficacy of protecting virus in elderly (18-month-old) mice. Such animals have a range of age-related immune deficiencies that parallel those found in people [6,10,12,38-44]. The data show that elderly mice are not only protected against a strong influenza type A challenge but also develop immunity to reinfection with the same challenge virus.

## Methods

### Viruses

Protecting virus 244/PR8 originally arose spontaneously after transfection of 293T cells with an infectious set of A/PR/8/34 plasmids [36]. 244/PR8 was amplified sequentially in MDCK cells and embryonated chicken's eggs, and purified by differential centrifugation through sucrose. Preparations were standardized at 2 × 10^5 ^HAU/ml and stored in liquid nitrogen. DI RNA 244 comprises 395 nucleotides and is derived from segment 1 RNA of PR8 by a single central deletion. Segment 1 encodes PB2, a component of the virion polymerase. 244 DI RNA comprises nucleotides 1-244 and 2191-2341 of the A/PR8 minus-sense segment 1 RNA, and retains the original termini and sequences essential for replication and encapsidation. Analysis by RT-PCR with primers specific for genome segment 1 showed that the 244 RNA was the major defective RNA present. Helper virus infectivity was eliminated by irradiating with UV for 40 seconds at 253.7 nm. This had little effect on the DI RNA because of its small UV-target size (395 nt) compared with the infectious viral genome (13,600 nt). Longer UV irradiation (8 minutes) destroys the protecting RNA but does not affect viral HA or neuraminidase (NA) activities [36], and provides a control for possible immune system-stimulation by antigen or receptor-blocking by the virus proteins [36]. The challenge virus, influenza A/WSN/40, was grown in embryonated chicken's eggs. The infectivity of clarified allantoic fluid was titrated in MDCK cells as focus-forming units (FFU). The dose required to cause disease in elderly mice was 4.3 × 10^3 ^FFU.

### Mice

We used inbred C3H/He-mg mice of both sexes which had once been part of a colony breeding programme. These were approximately 18 months of age with a mean weight of 45 g (range 36-52 g) at the time of inoculation. Compared with young adults they had increased body fat and thinner fur, were more poorly groomed, and considerably reduced activity levels, demonstrating the general demeanour of an elderly animal. Males were housed individually and most of the females as pairs, all in opaque plastic containers with a transparent lid. They thrived best in relatively small cages (35 × 14 × 13 cm) and were provided with food and water *ad lib*, paper bedding and cardboard tubes for recreation. Mice were treated with just one dose of protecting virus at the start of the study period. All inoculations were given as an intranasal drop to both nares under light ether anaesthesia. The dose of protecting virus comprised 12 μg virus protein. This relatively large dose [36] was chosen as insufficient elderly mice were available to permit titration. Individual mice were assessed clinically according to a scheme where each mouse is assigned a value from 1 to 5 according to increasing severity of disease, where 1 is well and 5 is dead, as detailed previously, and by mean group weight, on a daily basis [36]. All experiments were approved by the University of Warwick Ethics Committee and conform to the licence requirements of the UK Home Office.

## Results

### Protection by protecting virus 1 day before infectious challenge

**Figure 1:**
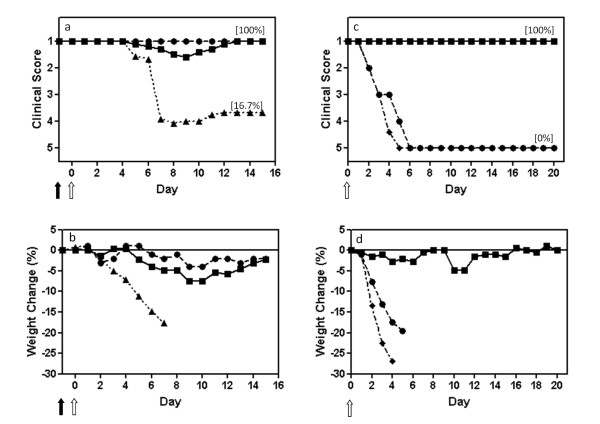
**A single dose of influenza A virus 244/PR8 given 1 day before infection protects elderly (18-month-old) mice from influenza, and these mice are then immune to further challenge**. Mice were anaesthetized and inoculated intranasally on day -1 with 244/PR8 or inactivated 244/PR8 (solid arrow), and were infected on day 0 (open arrow) with influenza A/WSN (panels a, b). Mice that had survived WSN challenge through treatment with protecting virus in a, b were challenged with a second high dose of WSN 3 weeks after the first infection (panels c, d) to establish their immune status. These mice were inoculated intranasally (open arrow) with a 500-fold higher dose of WSN than used in (a, b). (a, c) mean clinical disease assessment; (b, d) percentage weight change. ■, 12 μg 244/PR8 followed by WSN (n = 5); ▲, 12 μg inactivated 244/PR8 followed by WSN (n = 6); ●, 12 μg 244/PR8 followed by diluent (n = 2); ◆, naïve 5-week-old mice given only the high dose WSN challenge (n = 5). Mice were assessed clinically and weighed daily. Weights are not presented after 2 or more mice died. The percentage of mice surviving at the end of the study is in parenthesis. Data are representative of two separate experiments.

Mice were inoculated intranasally with a single dose of DI virus one day before intranasal infection with WSN. Figure 1a shows the clinical picture: mice given protecting virus alone or diluent (mock infected, data not shown) remained completely well. Those given UV-inactivated protecting virus before the infectious challenge became ill on day 5, progressing to serious disease that peaked on days 7-8. Most (67% or 4/6) were dead by day 8. In contrast, mice treated with active 244/PR8 before infection were all protected. Sixty percent (3/5) were protected completely, while 2/5 developed a mild illness, peaking on day 9 and from which they recovered completely. Comparison between the groups treated with active or inactive DI virus showed a highly significant statistical difference in the severity of disease (p = 0.0043) with a one tailed Mann-Whitney U test. Figure 1b shows that body weight in both 244/PR8 alone controls oscillated around the zero mark, as did mock infected controls (not shown). This contrasts with young adults where there is a steady weight gain. Mice given inactivated 244/PR8 before challenge lost weight at least 3 days ahead of clinical disease. Mice treated with 244/PR8 before infection showed only a small and transient weight loss, peaking at a maximum of 7% weight loss on days 9-10. They then regained the weight they had lost.

Having successfully protected elderly mice from acute influenza, we then determined their immune status. Animals that had received the active protecting virus and had survived the initial challenge infection were given a second WSN challenge at 3 weeks after the first infection to determine their immune status. This WSN dose (2.1 × 10^6 ^FFU/mouse) was 500-fold greater than the first dose and is high enough to overcome protection afforded by the initial 244/PR8 treatment. Thus any resulting protection is probably due to acquired immunity. Figure 1 (panels c and d) show that mice protected from the initial WSN challenge by administration of 244/PR8 (from Figure 1 (panels a and b)) showed no sign of clinical disease on rechallenge, whereas mice from the same experiment given 244/PR8 alone as a first treatment were all dead 6 days after challenge (Figure 1c). A virus control of naïve 5-week-old mice was also included to verify the potency of the challenge virus and these all died. Clearly 244/PR8 on its own did not stimulate a protective immunity. Since 244/PR8 cannot protect against this high dose of WSN, we conclude that protected mice had developed an adaptive immune response. Weight change data (Figure 1d) corroborated the clinical data with the 244/PR8-protected mice from Figure 1 (panels a and b) showing no significant weight change.

### Protection by protecting virus given 7 days before, and 1 day after, infectious challenge

**Figure 2:**
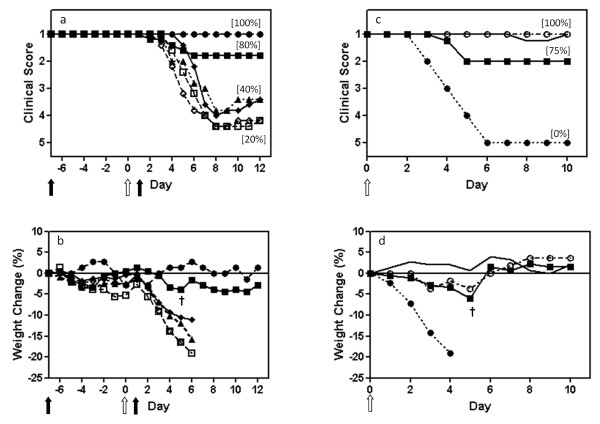
**244/PR8 given 7 days before infection protects elderly mice from influenza, and these mice are then immune to further challenge**. The experiment was set up as in Figure 1. Mice were treated intranasally with 244/PR8 at 7 days and 1 day before infection, and at 1 day after infection (solid arrows, panels a, b). Mice were infected with WSN on day 0 (open arrows). The immune status of survivors is investigated in panels c and d with a high dose WSN challenge. (a, c) show the mean clinical disease assessment, and (b, d) show the percentage weight change. Other information as in Figure 1. In panels a, b: ■, 12 μg 244/PR8 given 7 days before WSN (n = 5); □, 12 μg inactivated 244/PR8 given 7 days before WSN (n = 5); ◆, 12 μg 244/PR8 given 1 day after WSN (n = 5); ◊, 12 μg inactivated 244/PR8 given 1 day after WSN (n = 5); ▲, WSN only (n = 5); ●, 244/PR8 only (n = 2). In panels c, d: ■, 12 μg 244/PR8 given 7 days before WSN from (a, b) and rechallenged with WSN (n = 4); —, 12 μg 244/PR8 given 1 day before WSN from (a, b) (not shown) and rechallenged with WSN (n = 5); ●, saline inoculated controls only from (a, b) (not shown) challenged with WSN (n = 2); ○, non-infected controls (n = 2). The dagger in panels (b) and (c) indicates the death of a single mouse.

Mice were given a single intranasal dose of 244/PR8 at 7 days and 1 day prior to infection with WSN, and at 1 day after infection. Controls received the same dose of inactivated 244/PR8. Figure 2 (panels a and b) show that groups given inactivated 244/PR8 and WSN in the various timed combinations or WSN alone all became seriously ill, lost a major amount of weight, and suffered 40% death. In contrast, 80% (4/5) mice given 244/PR8 at 7 days before infection were completely protected with no significant weight loss. The non-protected mouse showed no delay in the onset of illness and no amelioration of clinical signs, suggesting that it may not have received a full dose of 244/PR8. The positive control group given 244/PR8 1 day before WSN were solidly protected (as in Figure 1 (panels a and b)) with only delayed slight, short lived clinical signs on days 7 and 8 in 2 of 5 mice, consistent with the data in Figure 1 panels a and b (data not shown). Comparison between the groups treated with active or inactive DI virus showed a highly significant statistical difference in the severity of disease (p = 0.0278) with a one tailed Mann-Whitney U test. 244/PR8 given one day after infection did not protect (p = 0.3452 comparing active and inactive DI treated mice and p = 0.4206 comparing active DI treated mice with infected untreated mice). Surviving mice were challenged with the very high dose of WSN as above to determine their immune status. Figure 2 (panels c and d) show that mice that survived the first infection through treatment with 244/PR8 at 1 day before infection were all solidly protected, as were most (75% or 3/4) of the group that originally received 244/PR8 at 7 days before infection. However, in such elderly mice it is expected that some mortality is associated with the age of the animals rather than infection. Age-matched control mice lost weight, became ill and died.

## Discussion and conclusions

We have previously shown that protecting virus prevents influenza in young adult mice. Protecting virus has two different modes of action. The observed cross-influenza A subtype antiviral activity of DI virus 244/PR8, coupled with its replication dependence on infectious virus and the common genetic system of all influenza A viruses, suggest that it acts at the level of genome competition [36]. Thus, it is likely that protecting virus can act against all influenza A viruses regardless of antigenicity, and indeed protecting virus prevents acute influenza A disease in SCID mice that have no adaptive immune response (submitted for publication). In addition, knock-out mice that have no functioning interferon type I response are also protected showing that this response is not essential for protection against challenge with homologous influenza A virus [45]. However, protecting virus stimulates interferon type I, and we believe this is responsible for heterologous protection *in vivo *from infection with pneumonia virus of mice (PVM), the acknowledged model for HRSV infection, and also influenza B virus. The importance of interferon type I in combatting these infections is shown by extensive reduction in protection against PVM and influenza B in mice lacking the interferon type I receptor [45]. In view of the potential role of the immune system in the mode of action of the protecting virus it is important to assess the efficacy of protecting virus in older mice which have a range of immune deficiencies reflecting those seen in the high risk elderly human population.

The data above demonstrate that a single dose of prophylactic 244/PR8 given up to 7 days prior to infection was highly effective in protecting elderly mice from a severe influenza challenge, and that most of the surviving mice were immune to reinfection. Protection at both levels operates despite the multiplicity of age-related immune deficiencies found in such elderly mice (see above). However, a single dose post-infection treatment was less effective than in young adult mice [36] suggesting that elderly mice are less able to respond to this type of therapy. We have not yet explored the nature of the resistance to reinfection in elderly mice, but parallel experiments show that treatment of young adult mice with protecting virus prevents the presentation of any sign of disease, but permits a reduced level of productive influenza lung infection. The residual level of virus replication is sufficient to elicit an immune response which can protect against subsequent challenge with an antigenically related virus [36]. It seems likely therefore that there is also enough infection in elderly mice treated with protecting virus to stimulate a virus-specific B cell- and T cell-mediated immunity. In conclusion, both elderly and young adult mice are capable of benefitting from treatment with protecting virus. Parallels with the elderly immunodeficient people suggest that protecting virus might be a useful additional weapon in the war against influenza.

## Competing interests

NJD and AJE hold patents relating to DI influenza virus. Otherwise the authors declare that they have no competing interests.

## Authors' contributions

NJD initiated the projected and carried it out with PDS. BM characterized the 244/PR8 and with ACM and AJE critiqued the work and contributed to the writing. This work has been read and approved by all authors.
